# Seasonal patterns of DOM molecules are linked to microbial functions in the oligotrophic ocean

**DOI:** 10.1128/msystems.01540-25

**Published:** 2025-12-30

**Authors:** Erin L. McParland, Fabian Wittmers, Luis M. Bolaños, Craig A. Carlson, Ruth Curry, Stephen J. Giovannoni, Michelle Michelsen, Rachel J. Parsons, Melissa C. Kido Soule, Gretchen J. Swarr, Ben Temperton, Kevin Vergin, Alexandra Z. Worden, Krista Longnecker, Elizabeth B. Kujawinski

**Affiliations:** 1 College of Earth, Ocean, and Atmospheric Sciences, Oregon State University 97120, Corvallis, Oregon, USA; 2 Department of Marine Chemistry and Geochemistry, Woods Hole Oceanographic Institution 830239 , Woods Hole, Massachusetts, USA; 3 Ocean EcoSystems Biology Unit, RD3, GEOMAR Helmholtz Centre for Ocean Research Kiel 28402, Kiel, Germany; 4 Marine Biological Laboratory 42700, Woods Hole, Massachusetts, USA; 5 Department of Biosciences, University of Exeter, Exeter, United Kingdom; 6 Department of Ecology, Evolution, and Marine Biology, Marine Science Institute, University of California 166653, Santa Barbara, California, USA; 7 Bermuda Institute of Ocean Sciences, Global Futures Laboratory, Arizona State University, St George's, Bermuda; 8 Department of Microbiology, Oregon State University 549630, Corvallis, Oregon, USA; 9 Microbial DNA Analytics, Phoenix, Oregon, USA; CNRS Delegation Alpes, Lyon, Rhône-Alpes, France

**Keywords:** dissolved organic matter (DOM), microbial communities, time-series, oligotrophic ocean

## Abstract

Hundreds of thousands of individual microbe-molecule interactions regulate the flux, transformation, and fate of carbon stored in the climatically important reservoir of marine dissolved organic matter (DOM). While marine microbial communities have been characterized at high resolution for over a decade, observations of the molecules cycled by the microbial-chemical network at similar resolution are limited. In addition, bulk characterizations of DOM can mask the complex network of interactions comprised of rich chemical diversities. Here, we present a three-year, depth-resolved, molecular time-series of DOM and prokaryoplankton at the Bermuda Atlantic Time-series Study (BATS) site. Both time-series exhibited seasonality that was compositionally distinct and primarily endemic to one sampling depth. We also putatively identified four exometabolites (gonyol, glucose-6-sulfate, succinate, and trehalose) that exhibit seasonal accumulation. We hypothesize these patterns result from environmental conditions that alter community composition on a seasonal timescale and thus shift the relative proportions of microbial functions that produce and consume the substrates. Critically, we observed the interannual composition of seasonal DOM molecules to be more stable than the taxonomy of the microbial community. This points to an important role of functional redundancy in regulating DOM composition. We tested this observation by querying metagenomes for pathways that utilize metabolic by-products putatively identified in the DOM time-series. We find that core microbial metabolisms, either those required by all or by a subset of marine microbes, are important predictors of DOM composition. The molecular-level characterization of DOM herein highlights the potential imprint of microbial activity on seasonal DOM composition.

**IMPORTANCE:**

Marine dissolved organic matter (DOM) is a major carbon reservoir that acts as a critical control on the Earth’s climate. DOM dynamics are largely regulated by a complex web of chemical-microbial interactions, but the mechanisms underpinning these processes are not well understood. In a three-year time-series, we found that the identity of the microbes is more likely to change between years than the composition of the DOM molecules. The taxonomic variability suggests that metabolisms shared across taxa, encoded by genes that conduct core microbial functions, are responsible for the more stable composition of DOM. While more than three decades of marine prokaryoplankton time-series are available, a similar reference for DOM molecules was missing. This time-series provides an improved understanding of the different responses of DOM molecules and microbes to seasonal environmental changes.

## INTRODUCTION

The ocean’s microbial food web is comprised of a taxonomically and functionally rich microbial community dependent on exchanges of thousands of chemically diverse organic molecules. Marine microbes fix, metabolize, and remineralize carbon and thus act as important source and sink mechanisms that regulate marine dissolved organic matter (DOM) flux and ultimately the Earth’s climate (1, 2). Both the chemical nature of DOM molecules (e.g., aromaticity, heteroatom content, size) and environmental conditions (e.g., community composition, nutrient dynamics, temperature) are proposed controls of DOM flux (3–5). However, the cryptic nature of the DOM-microbe network limits our ability to predict when changes in DOM composition will drive shifts in the microbial community or when microbial activity will alter DOM composition (6–8). This is further complicated by the vast diversity of both the DOM molecules and the microbial taxa. The chemical diversity of DOM is often categorized by a molecule’s reactivity, ranging from labile and semi-labile, which are the least abundant molecules due to their rapid recycling as microbial substrates, to refractory, which are the most abundant due to an accumulation of these molecules that evade microbial degradation (2). Similarly, the taxonomic diversity of the microbial community can be categorized by functional redundancies conferred through lateral or horizontal gene transfer, where many taxa harbor the same metabolic pathways responsible for producing or consuming the same DOM molecule (9). Disentangling the different controls of the DOM-microbe network is key to predicting changes in carbon flux in future oceans (10, 11).

As many biogeochemical functions mediated by marine microbes depend on the exchange of DOM molecules, microbial taxonomy and genome-encoded metabolisms should be, at least partially, predictive of DOM molecular composition. However, an important unknown remains with respect to the level of organization at which the DOM-microbe network should be defined. Previous laboratory and modeling experiments suggest that, in similar environmental settings, the metabolic functions of microbial communities are more predictable than taxonomic composition (12, 13), reflecting the fundamental nature of the core gene sets that encode the common metabolic pathways of a taxonomic or functional group. In contrast, a small number of differences in the genomes of closely related phylogenies of essential microbial community members underpin biogeochemically relevant niche differentiation, making taxonomic specificity at the strain level important for defining these groups (14, 15). Very recent marine metabolomics studies suggest that a limited number of marine metabolites are conserved across phylogenies, while others are taxonomically specific and even strain specific (16–18). Parameterizing the relationships between DOM molecules and microbial community functions requires experimental efforts that simultaneously probe both the marine microbial community and DOM molecules.

Seasonal environmental changes represent recurring disturbances that induce shifts in the taxonomy and function of microbial assemblages (19), thus creating a natural perturbation ideal for testing the resulting transformations in DOM composition. We leveraged these natural experiments of seasonal changes to assay the associated dynamics of DOM molecules, prokaryoplankton taxonomy, and prokaryoplankton functions in the northwestern Sargasso Sea at the Bermuda Atlantic Time-series Study (BATS) site. With more than three decades of sustained observations, the large-scale biogeochemical and physical fields of the seasonally oliogtrophic water column at the BATS site are well defined (20–23). In winter and early spring, the system experiences convective mixing as deep as 200–300 m, which delivers inorganic nutrients from depth and triggers an annual spring phytoplankton bloom. A quiescent and stratified period develops in late spring and persists into mid-autumn, during which the surface 100 m becomes highly oligotrophic. These dynamics also drive a seasonal cycle of bulk DOM, quantified as dissolved organic carbon (DOC), in the top 300 m at BATS (24–26). During the stratified season (typically May–October), DOM accumulates in the euphotic zone (0–120 m). A portion of the seasonally accumulated residual DOM is redistributed throughout the mixed layer and exported to the upper mesopelagic by deep convective overturning during the mixing season (typically January–March). Following re-stratification, the exported DOM becomes trapped in the mesopelagic, where it is subsequently remineralized by the resident microbial community.

Here, we present the *in situ* dynamics of a three-year depth-resolved time-series of both DOM molecules and free-living microbial prokaryoplankton sampled in parallel at the BATS site. We present the importance of seasonality in structuring the vertical stratification of both time-series and show that the interannual variability of prokaryoplankton taxonomy is greater than that of seasonally variable DOM composition using wavelet analysis. This suggests that microbial assemblage mechanisms are functionally redundant, allowing the resulting DOM biogeochemistry to remain consistent. Through targeted analyses of historical BATS metagenomes, we found a wide range in the degree of functional redundancy for enzymes responsible for producing and consuming seasonally-variant DOM molecules. Our work suggests that the flux of seasonally variable DOM molecules in the oligotrophic ocean will be more tightly linked to the presence of core metabolisms rather than the presence of specific microbial taxa.

## RESULTS AND DISCUSSION

In order to capture the major water column states of all four major seasons (summer stratified, fall transition, winter mixed, and spring transition) (Fig. S1) (27), we sampled the BATS site every two months for three years from July 2016 to July 2019 in the surface (1 m), upper euphotic zone (40 m), euphotic zone base (120 m), and upper mesopelagic zone (200 m) to produce parallel DOM and prokaryoplankton time-series. DOM was concentrated with solid-phase extraction and characterized with an untargeted liquid chromatography and ultrahigh resolution mass spectrometry approach, which provides a molecular overview of all detectable DOM. We detected 6,293 DOM features, each defined by a unique mass-to-charge ratio and retention time. These DOM features were pre-filtered for peak quality, blank contaminants, isotopes, and adducts, and thus represent, to the best of our ability, a unique set of molecules. We compared the DOM molecules with 3,158 prokaryoplankton amplicon sequence variants (ASVs), characterized using V1-V2 16S rRNA primers and pre-filtered to require presence in more than 5% of all time-series samples. We first present the seasonal and interannual temporal dynamics of the DOM molecules and prokaryoplankton ASVs. We then explore our hypothesis that core microbial metabolic functions play a role in regulating DOM composition by quantifying functional redundancy in publicly available metagenomes previously collected from surface waters at the BATS site.

### Wavelet analysis detects dominant periodicity in molecular time-series

Given the inherent challenges of comparing different data types across a time-series, we classified the temporal dynamics of DOM molecules and prokaryoplankton ASVs using wavelet analysis to decompose each time-series at every sampling depth into the frequency domain (Fig. S2) (28). Unlike clustering and correlation networks, which have been used to group unknown DOM molecules based on temporal or spatial patterns (29, 30), wavelet analysis provides additional insights that are concealed by these techniques, including the dominant periods of the time-series (e.g., 12 months) and the timing of a period maximum (e.g., peak in winter or summer). Wavelet analysis is also valuable for detecting periodicity variability and the resulting interannual differences (31, 32).

The dominant period of a DOM molecule or prokaryoplankton ASV within the time-series was assigned based on the highest median power, an estimate of best fit, across all calculated periods (2–12 months). The best fit was required to be significantly different from a null hypothesis test of “no periodicity” (median *P*-value *≤* 0.01), as might be expected for non-reactive DOM or persistent ASVs. We also required the time-series to have a relative standard deviation >25%, a threshold at which environmental variability is assumed to be greater than analytical variability, including inter-batch variability not removed by the batch correction, intra-batch variability from instrument performance, and variability induced from computational processing (33–35). Significant wavelets were found for 74% of DOM molecules (*n* = 4,679) and 67% of ASVs (*n* = 2,102) across the four sampling depths. The median powers ranged from 0.32 to 1.4, and the dominant periods ranged from 5 to 12 months. Almost all significant wavelets exhibited periods greater than 6 months, indicating that DOM molecules and prokaryoplankton ASVs with shorter frequency periods were more stochastic and too similar to random white noise to be significant. Higher-resolution sampling and a longer time-series would better detect patterns with shorter frequencies.

### Seasonal DOM molecules are depth-specific and differentiated by season

**Fig 1:**
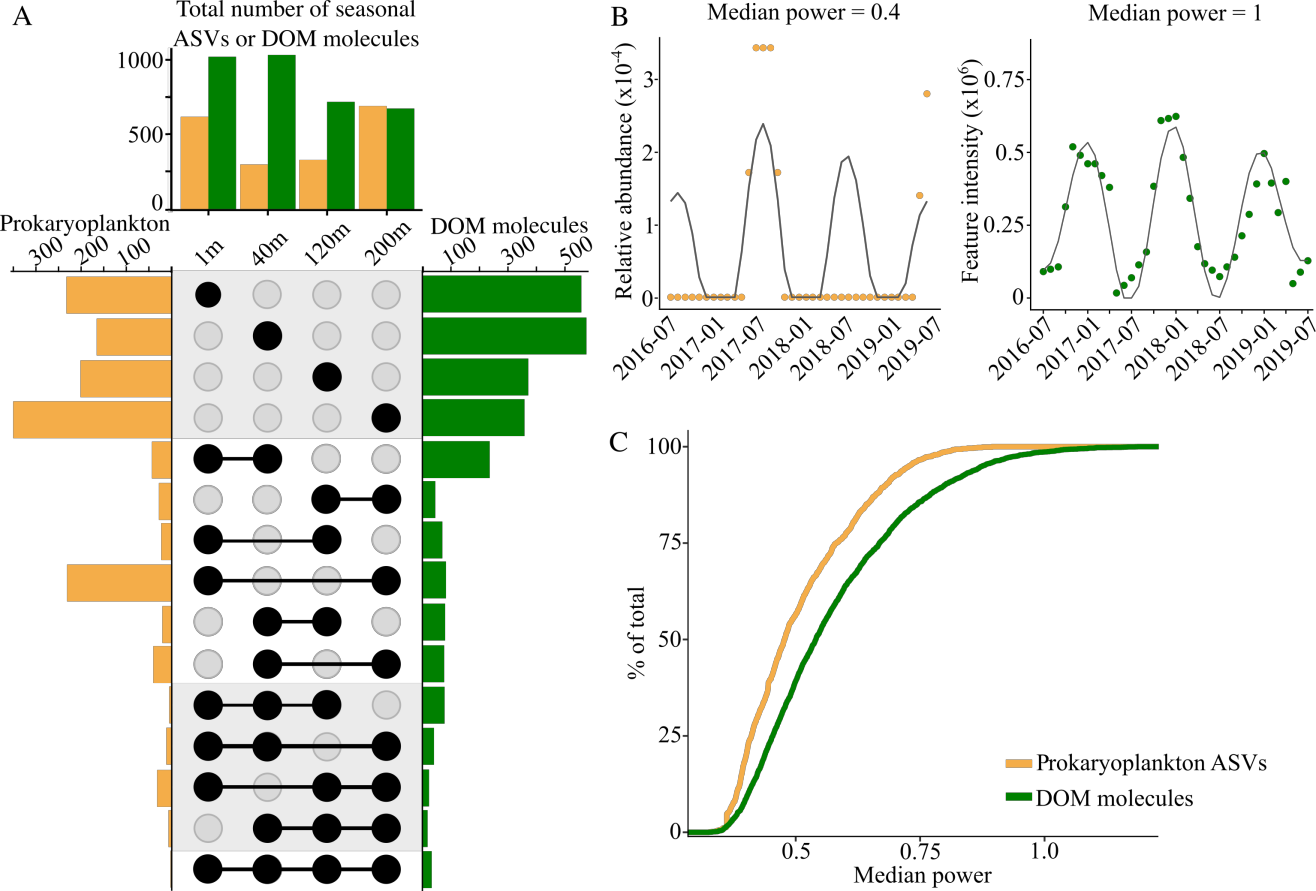
(**A**) Upset plot of seasonal prokaryoplankton ASVs (yellow) and DOM molecules (green). The top bar plot reflects the total number of seasonal ASVs and DOM molecules at each sampling depth. Side panel bars reflect the sum of ASVs or DOM moleculesthat exhibit seasonality at the respective sampling depth(s) as defined by the intersections in the black circles (i.e., rows 1–4 reflect ASVs and DOM molecules that were seasonal at only one depth, rows 5–9 represent seasonality at two depths, rows 10–14 represent seasonality at three depths, and row 15 represents seasonality at all four depths). (**B**) Examples of a seasonal ASV time-series with a low median power, driven by absences across the three years, and a seasonal DOM molecule time-series with a high median power, showing a repeated pattern across all three years. (**C**) Empirical cumulative distribution functions reflect the spread of median powers calculated for seasonal ASVs and DOM molecules.

At almost every sampling depth, the majority of both the DOM molecules and prokaryoplankton ASVs time-series exhibited a dominant period of 12 months, underscoring the important influence of seasonal environmental conditions at BATS. Approximately 40% of DOM molecules (*n* = 2,611) and ASVs (*n* = 1,385) exhibited seasonality, and we observed a majority of the seasonal periods at a single sampling depth for both the seasonal DOM molecules (*n* = 1,840) and seasonal ASVs (*n* = 862), indicating that the seasonal patterns are depth-specific (Fig. 1A). Almost twice as many DOM molecules exhibited seasonality at 1 m and 40 m compared to those at 120 m and 200 m (*n* = 1,098, 1,127, 700, and 665 seasonal DOM molecules, respectively). The greatest number of seasonal prokaryoplankton was found at 200 m (*n* = 576, 291, 313, and 698 seasonal ASVs at 1, 40, 120, and 200 m, respectively). While the spatiotemporal dynamics of the ocean’s microbiome are well described (36), this time-series highlights a similar, highly structured pattern of seasonal DOM molecules in the upper 200 m, which are predominantly endemic to specific sampling depths. Vertical stratification of the microbial community in the oligotrophic ocean presumably results from niche partitioning along nutrient and energy gradients (37–39), and these taxa are thought to release different DOM molecules (18, 40). Thus, the vertical niches of the microbial community would be associated with specific DOM molecular profiles, similar to the spatial stratification of seasonal DOM molecules observed in this time-series.

To predict the season in which a DOM molecule or prokaryoplankton ASV time-series with a dominant 12-month period reached its maximum, we reconstructed the time-series’ wavelets with a 12-month period and assigned the season based on the month in which the maximum of the 12-month period occurred. We found maxima for all four major seasons (summer stratified, fall transition, winter mixed, and spring transition) in the seasonal time-series of both DOM molecules and prokaryoplankton ASVs (Fig. S3). Most seasonal DOM molecules (33–49%) and seasonal ASVs (83–95%) peaked in the summer stratified season at every sampling depth, whereas the fewest peaked in the spring. The stratified periods encompass a large portion of the annual regimes at BATS, increasing the chances for our sampling to capture DOM molecules that exhibit a maximum during this period, whereas more frequent sampling is likely required to capture the short-lived spring transition (Fig. S1). While a majority of seasonal DOM molecules at 1 m peak during the summer stratified season, an almost equal number peak during the winter mixed season. Thus, the time-series captured not only the known trends of bulk DOM that have been previously observed at BATS, but also trends in opposition to the bulk DOM patterns.

Previous work describes DOM in the surface ocean that persists during summer stratification at BATS as semi-labile or semi-refractory DOM, which is not accessible to the surface microbial communities but is degraded by genetically distinct microbial communities at depth after physical export during convective winter mixing (4, 26, 41). A seasonal DOM molecule that reflects the patterns of bulk DOM would therefore be expected to (i) appear seasonally in both the upper and lower sampling depths and (ii) exhibit different temporal patterns at the sampling depths by peaking in the surface during summer stratification and in the deep after export by winter convective mixing. However, we observed very few seasonally variant DOM molecules in both the upper and lower sampling depths (Fig. 1A). Of the DOM molecules that exhibited seasonality in the surface and the deep, most exhibited the same seasonal maxima during summer stratification at both depths (Fig. S3). These unique temporal and spatial vertical dynamics indicate that the seasonal DOM molecules of this time-series are not just simply persistent molecules redistributed by convective mixing. We hypothesize that at least a portion of these DOM molecules could be introduced as by-products of the same metabolisms conducted across the different sampling depths or by other, more rapid export mechanisms, such as sinking particle solubilization or the vertical-migrating mesozooplankton shuttle (42, 43).

### The composition of seasonal DOM molecules is more stable than the taxonomy of prokaryoplankton

We next tested if the depth-structured seasonal patterns of DOM molecules and ASVs were consistent across the years. Wavelet analysis can uniquely capture localized temporal information, meaning that a 12-month period can be detected even if it is present in just one year of the three-year time-series. We quantified this interannual variability with the wavelet’s median power, an estimate of best fit (Fig. 1B). A high median power indicates the time-series fits well to the 12-month period, while a low median power indicates a poor fit, a signal that only appears in a portion of the three-year time-series, or both.

Across all sampling depths, the range of median powers was similar (0.3–1.3), but the distributions of median powers for the DOM and prokaryoplankton time-series were significantly different (Kolmogorov-Smirnov test: D = 0.2, *P*-value < 2e^-16^). The average median power of seasonal DOM molecules was greater than that of the seasonal ASVs (Fig. 1C), indicating that the composition of DOM molecules exhibits stronger recurring patterns between years at BATS.

The stable composition of seasonal DOM molecules across the three-year time-series, despite interannual changes in the prokaryoplankton community, suggests that some form of metabolic redundancy across the variable taxa promotes a recurring pattern of DOM composition. Other time-series studies found similar variability in the individual microbial taxa between years (44, 45), but the resulting feedback on seasonal DOM composition was previously unknown. This time-series indicates that the controls of taxonomic variability defined by the 16S rRNA gene differ from the controls of the dissolved molecules in the same environment, which challenges the utilization of highly resolved taxonomic information to predict DOM composition.

### Seasonal DOM molecules can be derived from biotic metabolisms

**Fig 2:**
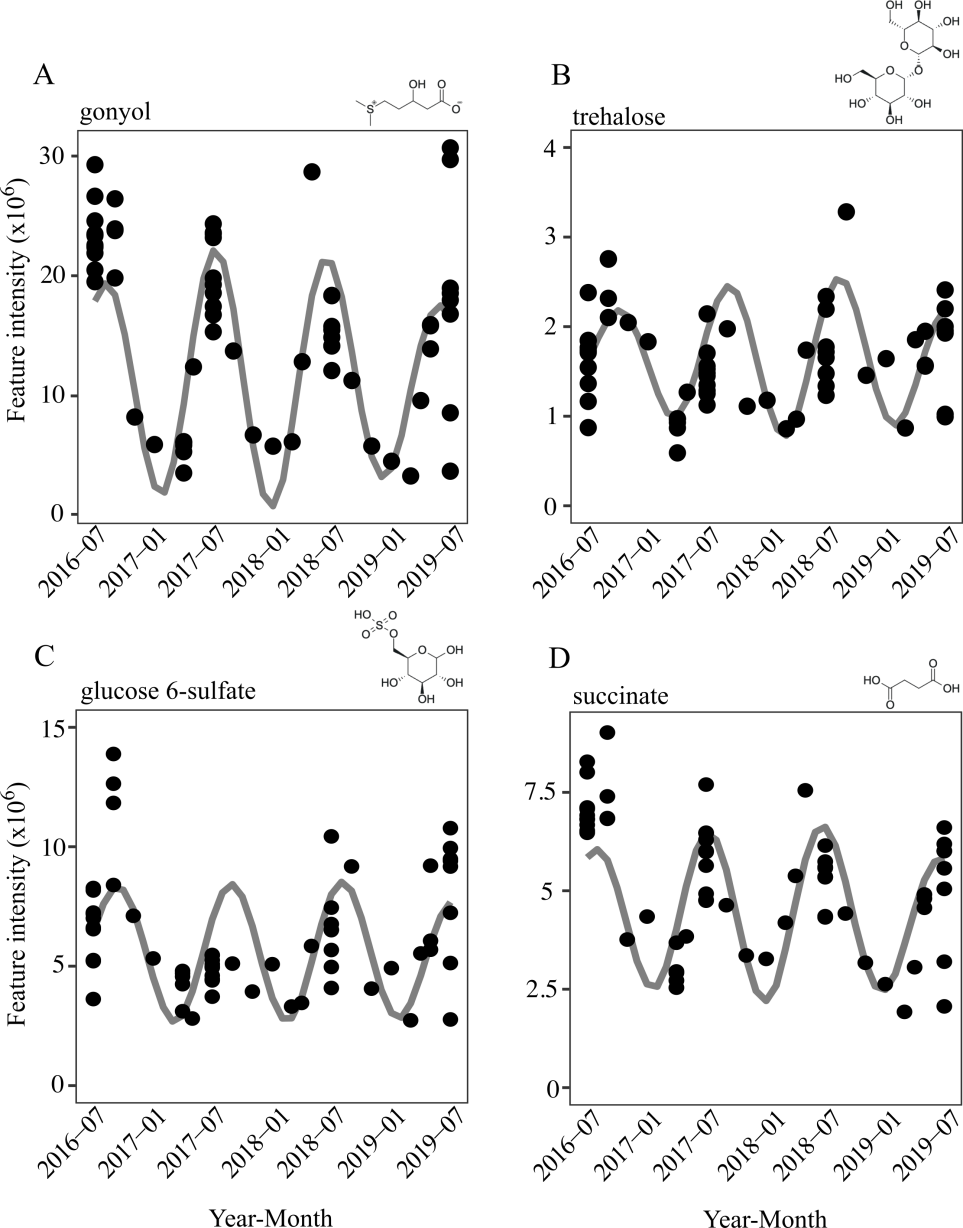
Three-year time-series of four putatively identified exometabolites at 1 m: (**A**) gonyol, (**B**) trehalose, (**C**) glucose 6-sulfate, and (**D**) succinate. The measured molecules’ intensities (arbitrary units) of the DOM molecule (black circle) are overlaid with the 12-month period wavelet reconstruction (gray line).

The molecular-level characterization of DOM allows us to examine the details of seasonal DOM composition that are obfuscated within the µM resolution of bulk DOC methods. Certainly, not all—and not even a majority—of the seasonal DOM molecules in the BATS time-series are expected to be derived from biological metabolisms. But still, previous studies identified important metabolic by-products, or exometabolites, using the same approach with solid-phase extraction of DOM (46–49). To further explore the role of exometabolites in the ocean’s DOM-microbe network, we highlight four putatively identified seasonal exometabolites, which were identified to the highest levels of confidence possible (level 1 or level 2) (50): gonyol, glucose 6-sulfate (or the isomer galactose 6-sulfate), trehalose, and succinate (Fig. 2; Fig. S4). These putatively identified exometabolites were of interest because of their potential for rapid microbial remineralization and their recurrent seasonality in the surface that peak in the summer stratified season. Based on previous work with structurally similar molecules, we assume that the four molecules have low extraction efficiencies (<1%) (51) and, therefore, must be present at high concentrations to be observable in this time-series.

Gonyol is a reduced organic sulfur molecule, structurally similar to the well-known metabolite dimethylsulfoniopropionate (DMSP), that is produced by eukaryotic taxa and rapidly metabolized by marine bacteria (17, 52, 53). The first quantification of gonyol in the dissolved phase was reported at low nM concentrations in the Pacific Ocean (54), and our time-series suggests that these concentrations would likely change seasonally. Glucose 6-sulfate is identical to the core metabolite glucose 6-phosphate, but the phosphate group is substituted for an oxidized sulfate group. To our knowledge, this is the first detection of glucose 6-sulfate in the oligotrophic ocean, and its potential sources or sinks remain an open question. Glucose 6-sulfate (or its isomer galactose 6-sulfate) could be a degradation product of presumably abundant, but poorly characterized, sulfated polysaccharides that comprise algal cell walls (55). Little is known about these large biopolymers in DOM. While most knowledge is derived from studies of macroalgae, including the brown alga *Sargassum* at BATS (56), microalgae and bacteria can also produce sulfated polysaccharides (57). A diverse suite of known sulfatases could remove the oxidized sulfate group for subsequent consumption of the remaining monosaccharide (41, 57, 58). Many intriguing questions remain regarding these two organic sulfur molecules, particularly in the context of the phosphorus-limited waters of the North Atlantic Ocean, where sulfur is known to be substituted into core biomolecules (59).

Trehalose and succinate are both metabolites for which little is known about their presence in marine DOM, but their genetic pathways are well characterized. Trehalose is a sugar that can be easily routed to glycolysis after breaking the disaccharide bond, but it has also been shown to be synthesized or retained as an osmolyte (60–63). Succinate is a dicarboxylic acid produced as an intermediate metabolic product in the citric acid cycle and glyoxylate pathway, making it a key part of core catabolic and anabolic pathways in marine microbes (64–66).

By tracking individual DOM molecules, we expected to observe patterns that diverge from bulk DOM. Based on the unique spatial and temporal stratification of seasonal DOM molecules, the time-series at BATS captured molecules with both abiotic and biotic control mechanisms. Many of the DOM molecules presumably follow the patterns of bulk DOM and persist due to inherent recalcitrance or environmental conditions that do not support microbial degradation. These seasonal molecules will be controlled by conservative dilution in the surface and physical export below the euphotic zone (24, 25). However, the seasonally variable DOM molecules in this time-series exhibit unique patterns that suggest we captured metabolic by-products rather than simply persistent molecules. Exometabolites are assumed to be labile molecules that turnover on hourly to daily timescales and, therefore, would lack seasonal accumulation patterns (2, 11). However, seasonality in the observed metabolic by-products may emerge from seasonal shifts in microbial community expression of production and consumption processes, which imprint a seasonal signature on top of shorter turnover fluxes. As in all environmental measurements, a single measurement is a snapshot of multiple timescales, including diel, seasonal, and interannual processes, as well as different spatial scales of allochthonous and autochthonous production. The DOM time-series represents standing stocks of molecules comprising the DOM reservoir, which are the culmination of many different amplitudes of environmental variability.

### Core metabolisms are important predictors of DOM composition

Marine microbial communities are extremely diverse but share core genes that confer the same functions and depend on the same metabolic reactants (36). This functional redundancy is important for maintaining microbial ecosystem functions when communities change (67, 68). We hypothesized that recurring patterns of the same DOM molecules (Fig. 1C) result from interannual changes in the prokaryoplankton community, defined by taxonomic variability but convergent functionality. We tested this hypothesis by quantifying the functional redundancies and taxonomies of metabolic reactions requiring the putatively identified exometabolites in historical metagenomes. These exometabolites are examples of the seasonal DOM molecules observed in the BATS time-series that exhibited consistent interannual seasonal patterns, but also their utilization is expected to differ significantly across the microbial community. Succinate is broadly used as part of the citric acid cycle, whereas trehalose is used more narrowly as a carbon substrate or osmolyte. The biosynthesis and catabolism of gonyol and glucose 6-sulfate are not known, and thus could not provide estimates of functional redundancy.

**Fig 3:**
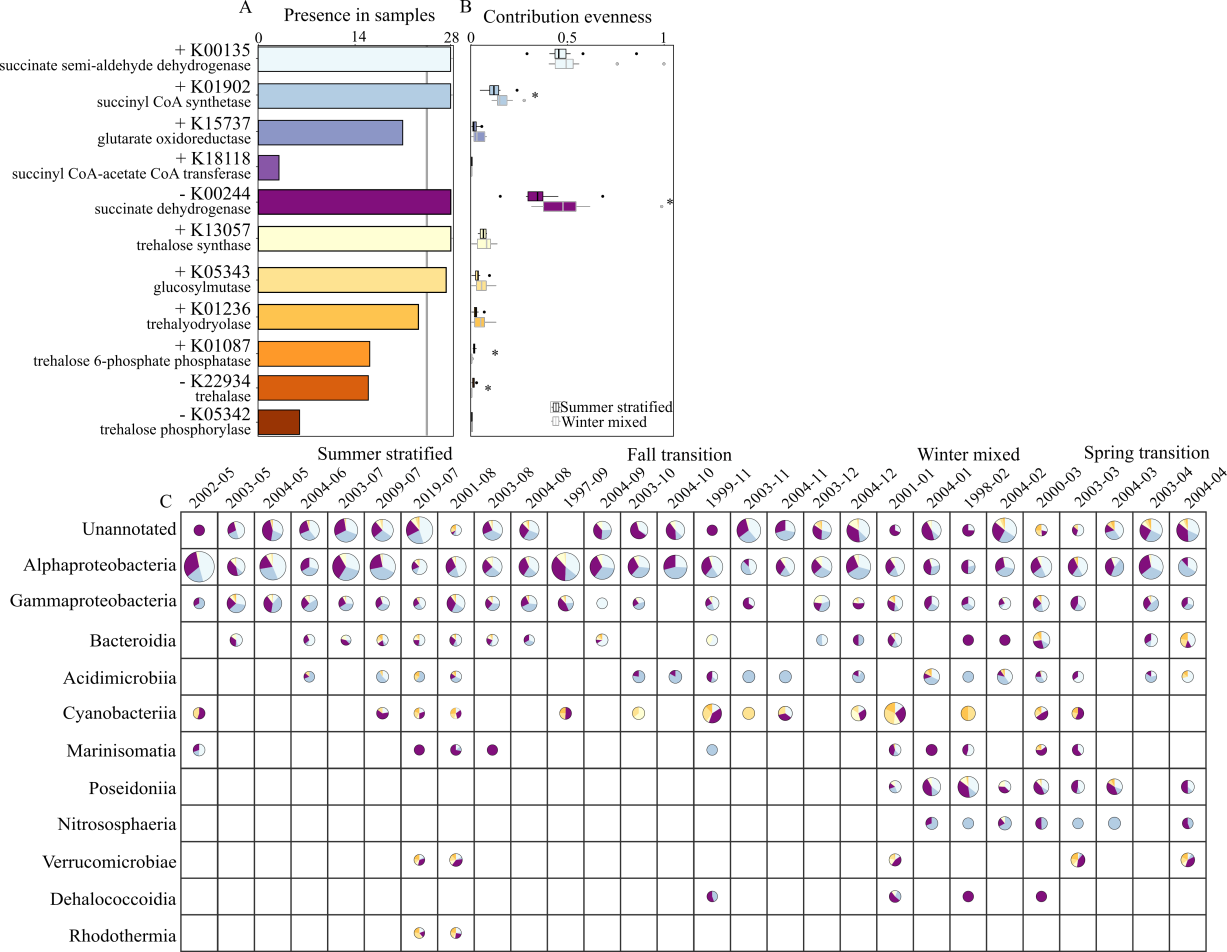
(**A**) Presence of KOs (KEGG orthologies) that utilize succinate (cool colors) or trehalose (warm colors) as a product or reactant in surface metagenomes at BATS. + indicates metabolite is product. − indicates metabolite is reactant. KOs present in >85% of samples (gray vertical line) are assumed to be core genes. (**B**) Functional redundancy of the same KOs as estimated with the contribution evenness (CE) metric. Higher CE indicates higher functional redundancy. The outline of the boxplot indicates CE values of samples collected in the summer stratified season (black outline) or the winter mixed season (gray outline). A star indicates that CE was significantly different between seasons (Wilcoxon rank sum test, *P*-value ≤ 0.1). (**C**) The functional taxonomy of the same KOs depicted in pie charts for every sample (columns) organized by season and the most commonly annotated (present in *≥*2 samples) taxonomic group (rows). Each pie is divided by the relative contribution of the respective taxonomic group to each of the six succinate or trehalose core KOs (K00135, K01902, K00244, K13057, K0543, K01236) based on total RPKM (reads per kilobase per million mapped reads) in a given sample. KO colors are the same as shown in panel **A**. The pies are scaled based on the total relative contribution of each taxonomic group to the sample.

We searched for functional orthologs (KOs) that use trehalose (*n* = 6 KOs) or succinate (*n* = 5 KOs) as a product or reactant in 22 years (1997–2019) of publicly available surface ocean metagenomes collected at the BATS site (*n* = 28 samples) (Tables S3 and S4). These samples were not uniformly collected, but they capture all four seasons of the BATS physical framework (sample numbers from each season: summer stratified [*n* = 12], fall transition [5], winter mixed [9], and spring transition [2]). A subset of KOs for both succinate and trehalose was present in 100% and >85% of all metagenome samples, respectively, and were assumed to be core genes common to the surface microbial community (Fig. 3A). We estimated functional redundancy using the contribution evenness (CE) metric based on the abundances of trehalose and succinate KO (69). Unlike traditional metrics of functional redundancy, which are based on niche space and are not easily translated to microbial communities, the CE metric was developed to quantify the redundancies of specific nucleic acid sequences as microbial traits. CE ranges from no redundancy (CE = 0) to absolute redundancy (CE = 1), which would indicate that all community members contribute equally to the presence of the KO of interest. The median CE across all samples ranged from 0 to 0.5 for succinate-related KOs, and the maximum CE was 1.0 (both K00135 and K00244) (Fig. 3B). These high CE values reflect the ubiquity of the citric acid cycle, and succinate utilization can be defined as a broad-type redundancy (70). In contrast, the median CE across all samples ranged from 0 to 0.1 for trehalose-related KOs, and the maximum CE was 0.13 (K13057). These lower CE values reflect a known narrower utilization of trehalose in marine microbial communities (71) and can be defined as a specific-type redundancy used by a subset of the community (70). The CE of succinate-related KOs was overall significantly greater than that of trehalose-related KOs (Wilcoxon rank sum test *P ≤* 0.01), indicating greater redundancy in succinate metabolism.

Functional taxonomy of these KOs further reflected the differentiation of the microbial community’s ability to utilize succinate or trehalose (Fig. 3C). Core genes encoding for enzymes required for the citric acid cycle (K00135, K01902, and K00244) were most commonly annotated as Alphaproteobacteria and Gammaproteobacteria, specifically *Pelagibacter* and SAR86, which together accounted for *≥* 67% and *≥* 15% of the sum of annotated RPKM (reads per kilobase per million mapped reads) for each core KO. As these are dominant groups in the surface ocean at BATS, it is not surprising that they dominate the taxonomy of KOs required for the widely used citric acid cycle (72, 73). In contrast, the functional taxonomy of core KOs encoding for enzymes that synthesize trehalose (K13057, K05343, and K01236) was more specific. The Alphaproteobacteria were the most commonly annotated taxonomic contributors of the trehalose synthase K13057, accounting for 64% of the sum of annotated RPKM, whereas the other two core trehalose genes (K01236 and K05343) were most commonly annotated as Cyanobacteria or Bacteroidia, which accounted for >31% and >15%, respectively, of the sum of annotated RPKM for each KO. Although the extent of functional redundancy and functional taxonomy differs between succinate and trehalose, both metabolites are required reactants or products of core genes in the BATS microbial community and also exhibit very similar patterns in the DOM time-series.

Here we describe a framework of the potential seasonal and interannual trends of the DOM-microbe network, based on the observations from the time-series as well as the functional redundancies and taxonomies in the historical BATS metagenomes. Between seasons, we observed apparent shifts in the standing stocks of seasonal DOM molecules. For seasonally variable DOM molecules with biotic controls, a shift in either the functional redundancy (Fig. 3B) or the dominant functional taxonomy (Fig. 3C) could generate the observed patterns. CE of some succinate and trehalose genes was not significantly different between the summer stratified and winter mixed seasons, but there was a general taxonomic shift in the major contributors. For example, CE of the most redundant succinate KO (K00135) was not significantly different between seasons, but the dominant functional taxonomy shifted from Alphaproteobacteria in the summer stratified season to Poseidoniia (Thalassarchaeaceae) in the winter mixed season. In contrast, the CE of other genes, such as the second most redundant succinate KO (K00244), was significantly enhanced or suppressed between the summer stratified and winter mixed seasons, indicating that the community’s total potential to produce or consume the exometabolite changed seasonally. Both types of seasonal microbial shifts could alter standing stocks of DOM due to changes in the enzymatic efficiency associated with the taxonomic shifts, changes in the total number of taxa capable of interacting with the exometabolite, or changes in metabolism in response to seasonal shifts of environmental drivers.

Between years, we observed significant taxonomic variability of the prokaryoplankton ASVs, but less variability in DOM molecules of our time-series. Interestingly, both succinate and trehalose exhibited seasonal patterns in DOM that remained consistent across all three years, but their functional redundancies and taxonomies differed. Ecological theory suggests that, due to its lower functional redundancy, trehalose is more susceptible to variability in microbial community compositions (68). We suggest that the role of a molecule in reactions encoded by core genes of a microbial community is more important than the degree of functional redundancy. In other words, even if a core gene is essential to the function of just a subset of community members, the associated molecule will still be regularly exchanged through the DOM-microbe network.

### The current challenges of molecular time-series

The DOM time-series at the BATS site provides some of the first molecular-level insights into the variability of DOM molecules on seasonal and interannual timescales in parallel with the microbial community. Our understanding of marine DOM dynamics and composition is limited to what we can detect. A benefit of untargeted tools is the potential to identify previously unrecognized DOM molecules, but there are also many important nuances required for interpretation of these data. For example, we putatively identified gonyol and glucose 6-sulfate in DOM as ecologically relevant molecules, which both lack annotated pathways for synthesis and degradation and thus are not detectable by other ‘omics approaches. To our knowledge, this is the first description of glucose 6-sulfate in marine DOM, which is an intriguing potential analog of glucose 6-phosphate, but future work is required to uncover its source and chromatographically separate its potential stereoisomer galactose 6-sulfate. Another critical consideration for interpreting this time-series is the reliance on solid phase extraction, which is currently the most common approach to DOM isolation, but is known to select for a subset of DOM. Solid-phase extracted DOM is biased toward more recalcitrant-like properties defined by smaller sizes, lower C:N ratio, and limited bioavailability (74–77). Thus, it was surprising, although not unprecedented (49), to observe the four putatively identified exometabolites which have very different chemical properties. The detection of the four putatively identified exometabolites highlights their importance in seasonal marine DOM dynamics and identifies molecules for validation in future work using targeted extraction techniques (e.g., [54, 78]). Finally, an intriguing challenge presented by the thousands of unknown DOM molecules is differentiating, at a molecular level, those that are biologically active from the background reservoir of refractory DOM molecules. Similar challenges exist for other molecular, biogeochemical measures. For example, it has been estimated that even in the surface ocean, only ~55% of the total detected taxa (DNA) are active community members (RNA) (79). Future studies could consider leveraging tools that can “tag” biologically sourced molecules (80). The field of environmental metabolomics is relatively young compared to other molecular tools, and this time-series represents an important contribution to our understanding of marine DOM that expands baseline knowledge of different temporal and spatial variabilities. Community-wide efforts are needed to address these challenges and fully maximize the tools’ use in the environment. These include regular collection of *in situ* samples, expansion of data repositories to increase annotation rates, standardized best practices for data processing (81), and the development of alternative extraction techniques.

### Conclusions

Disentangling how the reservoir of marine microbial diversity translates into a similarly diverse pool of DOM molecules is critical for a better understanding of carbon cycling in the ocean. In an environment that has already experienced 1.2°C of warming (23), resolving these baseline processes is essential in order to predict future changes in the ocean’s organic carbon cycle. We demonstrate that metabolic functions, rather than taxonomic identity, are likely better predictors of seasonal DOM composition. Despite similar complexities with respect to composition, we found that the mechanisms responsible for driving prokaryoplankton taxonomy and DOM molecules differ. This work suggests that models predicting biotically regulated DOM flux should incorporate core metabolic pathways essential for community function, either by all or a portion of the microbial community.

Despite the observed taxonomic variability of the prokaryoplankton community at the BATS site during the three-year time-series, the changes were not enough to influence the composition of the resulting DOM biogeochemistry. This buffer of functional redundancy overlaid on taxonomic variability will play an important role in future oceans. How much can microbial taxonomy change, though, before the presence of these core metabolisms is altered? As anthropogenic carbon emissions alter the ocean’s temperature, pH, and nutrient status, microbial communities are predicted to shift and evolve in response and, in some cases, may do so abruptly (82–84). If the shifts are severe enough, these stressors will eventually alter functional redundancies and subsequently DOM biogeochemistry. This work presents a major advance in our understanding of variability and composition of the individual molecules comprising DOM, as well as important avenues of research for predicting the resulting carbon flux. The seasonal patterns of DOM molecules represent snapshots of standing stocks, and future studies that emphasize rate measurements will be essential. Continuing to resolve the influences of the microbial loop’s functional redundancy and core metabolisms on DOM biogeochemistry will be critical for predicting changes in ecosystem function, particularly heterotrophic carbon remineralization, in future oceans.

## MATERIALS AND METHODS

### DOM sample collection and extraction

Samples were collected aboard the R/V *Atlantic Explorer* every two months from fixed depths (1 m, 40 m, 120 m, 200 m) at or in the vicinity of the BATS site from July 2016 to July 2019. During July field campaigns, samples were collected from the four sampling depths every 6 h for 72 h. During all other sampling events, one sample per depth was collected, primarily between 05:00 and 10:00 local time. The chosen sampling depths are important sites of seasonal changes at BATS, defined by a physical framework of biogeochemical water column properties (Fig. S1) (27). We sampled all four major seasons (summer stratified, fall transition, winter mixed, and spring transition) every year, with the exception of the spring transition in 2017, which is a short-lived period that likely occurred between our April and May 2017 sampling. Four liters of whole seawater was collected directly from Niskin bottles into polycarbonate or Teflon bottles and subsequently filtered through a 47 mm, 0.2 µm Omnipore PTFE filter (Millipore, MA USA) using PFA in-line filter holders (Advantec, CA USA), Nalgene FEP tubing (ThermoFisher Scientific, MA, USA), and a peristaltic pump (85). Four liters of onboard Milli-Q water was filtered in the same manner for process blanks. The filtrate was acidified to a pH of 2–3 with OmniTrace HCl and extracted via solid-phase extraction with styrene-divinylbenzene polymer columns (1 g, 6 mL Bond Elut PPL, Agilent, CA, USA). The resins were washed with one cartridge volume (~6 mL) of LC-MS grade methanol. The acidified filtrate was loaded onto the resin via an extraction manifold using Nalgene FEP tubing and vacuum pressure. After sample loading, the resins were rinsed four times with 0.01 M HCl, dried for 5 min, and eluted with one cartridge volume of LC-MS methanol (74, 86). Although bulk DOC concentrations at BATS change seasonally (26), we estimate that even at the highest surface concentrations of ~70 µM DOC, we loaded an order of magnitude less carbon (4 L × 70 µM DOC = 3.36 mg DOC) onto the resin than its maximum capacity (5% of bedmass (1 g) = 50 mg DOC). Sample elutions were evaporated to near dryness and reconstituted in Milli-Q water with stable-isotope-labeled internal injection standards (Table S1). A pooled sample was created with an aliquot of every sample. All plasticware was sequentially rinsed three times with 10% HCl acid-washed, Milli-Q, and finally with the seawater sample. All glassware was combusted at 450°C for 4.5 h.

### UHPLC-ESI-MS/MS

Separation was performed with a reverse-phase Acquity HSS T3 column (2.1 × 100 mm, 1.8 µm), equipped with a Vanguard pre-column (Waters, MA, USA), on an ultrahigh-performance liquid chromatography system (Vanquish UHPLC, Thermo Scientific) coupled with an Orbitrap Fusion Lumos Tribrid mass spectrometer (Thermo Fisher Scientific). Column temperature was held at 40°C. The column was eluted at 0.5 mL/min with a combination of solvents: (A) 0.1% formic acid in water and (B) 0.1% formic acid in acetonitrile. The chromatographic gradient was as follows: 1% B (1 min), 15% B (1–3 min), 50% B (3–6 min), 95% B (6–9 min), and 95% B (10 min). The column was re-equilibrated with 1% B (2 min) between injections. The autosampler was set to 4°C, and injection volumes were 5 µL. The source settings were set as follows: electrospray voltage was 2,600 V for negative mode and 3,600 V for positive mode; source sheath gas was set to 55 and auxiliary gas was to 20 (arbitrary units); the heated capillary temperature was 350°C; and the vaporizer temperature was 400°C. MS data were collected using the Orbitrap analyzer with a mass resolution of 120,000 FWHM at m/z 200. The automatic gain control (AGC) target was set to 4e5, the maximum injection time was 50 ms, and the scan range was 100–1,000 m/z. Internal mass calibration of the Orbitrap analyzer was used to improve mass accuracy of the MS scan. Data-dependent MS/MS data were acquired in the Orbitrap analyzer using higher energy collisional dissociation (HCD) with a normalized collision energy of 35% and a mass resolution of 7,500. The AGC target value for fragmentation spectra was 5e4, and the intensity threshold was 2e4. Cycle time was set to 0.6 s. Precursor selection was performed within the quadrupole using a 1 m/z isolation window. Dynamic exclusion was enabled, with a three-second exclusion duration after *n* = 1. The sample set (*n* = 374) was randomized across five batches. The pooled sample was used for column conditioning and was also injected after every five samples and at the end of each sequence, followed by process blanks and Milli-Q blanks. Batches were run in both positive and negative ionization mode. All data were collected in profile mode.

Large LC-MS/MS experiments are prone to retention time drift, contamination, and carryover between samples (87). We mitigated these issues as follows: LC-MS sequences were limited to 105 injections (~18 h), the internal mass calibration was enabled (equivalent to a lock mass correction), the column was re-equilibrated after every injection, sample order was randomized, the ESI probe was cleaned between batches, multiple stable-isotope-labeled internal injection standards were added to all samples (Table S1), and a pool QC sample was run after every *n* = 5 samples. After running the experiment, we discovered that the pool sample was sub-sampled too many times, resulting in a linear decrease of the pool samples’ total ion chromatogram intensities across each batch. This prevented us from using traditional metrics for correcting variance with pool samples (87). However, one pool sample was aliquoted per batch, and a variability metric was calculated by comparing the first injection of each batch (*n* = 5).

### XCMS and CAMERA workflow

Raw data files were converted to mzML format using msConvert (88) and transferred to a high-performance computing cluster for processing with R (v 4.0.1). XCMS (v 3.10.2) was used for peak picking each sample and grouping shared peaks into a single feature (89). Peak-picking was performed using the CentWave algorithm with the following parameters: noise = 100, peak-width = 3-14, ppm = 15, prescan = 3, preintensity = 5e4, snthresh = 0, integrate = 2, mzdiff = −0.005, extendLengthMSW = TRUE, fitgauss = FALSE, and firstBaselineCheck = FALSE. Replicate picked peaks were merged using refineChromPeaks (MergeNeighboringPeaks Param: expandRt = 0, expandMz = 0, ppm = 5, minProp = 0.75). Peaks were filtered based on peak quality by requiring a peak width less than 15 s and with a custom R script that screened for Gaussian fits in peak shapes (correlation value > 0.6 and a *P*-value < 0.075). Retention times were adjusted using Orbiwarp (binSize = 0.1) based on the center sample (90). Correspondence between the peaks was conducted using the peak density method (bw = 0.7, binSize = 0.0005) (89). These parameters were optimized based on internal injection standards and manual checks. We did not use the fillChromPeaks, as it primarily resulted in the integration of noise. Feature values were integrated by the ‘maxint’ method. CAMERA was used to identify isotopes and adducts by grouping features based on retention time to create pseudospectra (perfwhm = 0.5), identifying ^13^C isotopologues (ppm = 3, mzabs = 0.01), and grouping based on correlations of intensity, extracted ion chromatograms, and isotopes (corr_eic_th = 0.9, cor_exp_th = 0.8, pval = 0.05) (91). XCMS was also used to produce MGF files (consensus spectra and maximum total ion current spectra).

### Feature filtering

The XCMS and CAMERA workflow resulted in *n* = 153,360 features in positive mode and *n* = 117,079 features in negative mode. Feature intensities were batch corrected using the BatchCorrMetabolomics package (v 0.1.14) with robust least-squares regression (92).

There is no single solution for peak picking data analysis, meaning that even after optimization, a single set of parameters does not resolve all desired features correctly. We opted initially for a lenient set of parameters, and then used stringent filtration practices to remove the noisy undesired peaks. Features were filtered based on results from CAMERA to remove identified isotopologues and adducts (91).

Features were filtered using Milli-Q and process blanks with a data-adaptive method (93). The mean log abundance across samples and blanks was calculated for each feature and subsequently binned into 20, 40, 60, and 80th quantiles. For each bin, a threshold was calculated based on the 25th quartile of the difference between the mean log abundances of samples and blanks that were less than 0. The difference for all features in a given bin was required to be greater than the absolute value of this threshold. Features were filtered to require their grouped peaks to have a range in median retention times of less than 5 s. Features were filtered to require their detection in >50% of all samples. If a feature was detected in the pool sample, it was required to have a relative standard deviation <20%, as calculated based on the intensity across the first pool sample injected in each batch (*n* = 5). The filtered features represent 4% of the original features output by our XCMS workflow. The remaining features represent, to the best of our ability, unique molecules. Presented intensities are unitless and reflect the integration of all ion counts associated with a given feature’s m/z ratio, bounded by the retention time window.

### Metabolite identification

MGF files and abundance tables from XCMS were submitted to the Global Natural Products Social (GNPS) Molecular Networking infrastructure for feature-based molecular networking (94). The putative identifications had m/z matches to expected masses within ± 1 ppm (Table S2) and high cosine scores matching reference spectra (Fig. S4). The identifications were originally made by GNPS and subsequently confirmed with authentic standards, when available. Based on confidence levels defined by the Metabolomics Standards Initiative (50), succinate, trehalose, and gonyol were identified to the highest level possible (level 1) using standards analyzed on the same analytical platform used for the untargeted analysis. Glucose 6-sulfate was identified to the second-highest confidence level (level 2) because, to the best of our knowledge, an authentic standard for this compound does not exist. The putative identification was instead made based on a match to the MS2 reference spectrum of the almost identical compound, glucose 6-phosphate, where the exact mass difference between the two precursor masses (0.009 m/z) matches the expected mass difference between glucose 6-sulfate and glucose 6-phosphate (0.0095 m/z). A dominant MS2 fragment had m/z 96.959 (HSO_4_-); in comparison, a phosphate-containing fragment would have a mass of m/z 96.969 (H_2_PO_4_). With the chromatography used, we cannot rule out that the putatively identified glucose 6-sulfate could instead be the isomer galactose 6-sulfate.

### Microbial community, 16S rRNA amplicon sequencing, and data filtering

Samples for 16S V1-V2 ASVs were collected as described in (26). Only samples collected at 1 m, 40 m, 120 m, and 200 m were presented. Briefly, 4 L of seawater were filtered onto 0.2 µm Sterivex and stored at −80°C. DNA was extracted with a phenol-chloroform protocol (37). The V1-V2 16S rRNA hypervariable region was amplified with primers 27F (5′-AGAGTTTGATCNTGGCTCAG-3′) and 338RPL (5′-GCWGCCWCCCGTAGGWGT-3′). Amplicon libraries were built using the Nextera XT Index Kit (Illumina Inc.) and sequenced using the Illumina MiSeq platform (reagent kit v2; 2 × 250 PE) at the Center for Quantitative Life Sciences, Oregon State University. Raw amplicon data sets were processed as in (32) using Dada2 v1.18 (95) with the following filtering parameters: maxEE = (2,2), truncQ = 2, minLen = 190, truncLen = (220, 190), and maxN = 0. Samples from the same sequencing run were processed together to accurately estimate the error frequency. Potential chimeras were removed using the removeChimeraDenovo command. Taxonomic assignment was performed with the assignTaxonomy command and the SILVA non-redundant database V.123 (96). Generated ASV and taxonomic tables were analyzed using phyloseq v1.34 (97). ASVs were presented as relative abundances, normalized to the total counts of all ASVs in a respective sample. ASVs were required to be detected in ≥ 5% of all samples. This yielded *n* = 3,158 ASVs.

### Wavelet analysis

Wavelet analysis was used to decompose the exometabolome and ASV time-series using the R package WaveletComp (28) (Fig. S2). Wavelet analysis requires a uniform grid. Most of the time-series was sampled in odd months, except for the samples collected in April. We interpolated between months to create a monthly time-series that allowed us to incorporate the April data. This also avoided any distortion to the wavelet analysis, which is sensitive to time-series length. In months where more than one sample was collected (primarily July diel campaigns), we used the average feature intensity as the representative value.

Significance was assessed with the null hypothesis of white noise, and 1,000 permutations were calculated for each time-series. The best fit was required to be significantly different from a null hypothesis test of “no periodicity” (median *P*-value *≤* 0.01). Similar trends of DOM molecules were observed in both ionization modes, and therefore, only positive mode results were presented. We also required all time-series classified as having significant wavelets to have a relative standard deviation that represents a threshold for which environmental variability should be greater than analytical variability (>25%) (33).

### Functional redundancy and functional taxonomy

We searched publicly available surface sample metagenomes collected at BATS from 1997 to 2019 (Table S3). HMMER (v 3.3.1, hmmer.org) searches were conducted with HMM profiles previously created by KofamScan (98). KO numbers were collected based on the analysis of KEGG Pathways (99) to identify key enzymatic reactions required to conduct pathways that result in the production or consumption of trehalose and succinate (Table S4). Multiple KOs can encode for the same metabolic transformation, and for brevity, we present the most redundant KO only. In addition, single-copy marker genes (SCMG) were searched to estimate sample richness (K01409, K01869, K01873, K01875, K01883, K01887, K01889, K03106, K03110, K06942). KO HMM results were either filtered with an e-value of 1 × 10^–10^, or, for SCMG KOs, filtered based on threshold scores defined by KofamScan. The taxonomy of metabolic KO genes was assigned using the contig level taxonomy annotations from MDMcleaner (v 0.8.2) ‘clean’ output with ‘-fast_run’ settings (100).

Presence was calculated as the number of contigs assigned to a metabolic KO. The metric of contribution evenness CE was calculated as an estimate of metabolic redundancy (69). CE ranges from no redundancy (CE = 0), indicating that only one community member in the sample harbors the gene, to absolute redundancy (CE = 1), indicating that all community members contribute equally to the gene’s presence. As expected, and based on the increase in sequencing power in the last 20 years, sequencing depths varied by orders of magnitude across the different metagenomes (Fig. S5). Despite the order of magnitude differences in sequencing depth, there was a log-log linear relationship between the richness of SCMG KOs and the succinate and trehalose KOs. CE accounts for these differences by normalizing KO abundances to total species richness as estimated by the presence of universal single-copy marker genes, which are assumed to occur once in each genome.

Samples richness was calculated based on the number of contigs encoding a SCMG. All figures were created using ggplot2 (v 3.4.3) and curated with Inkscape (v 1.2.2).

## Data Availability

Metabolomics data, including raw files, mzML files, and feature tables, are deposited at MetaboLights under study accession number MTBLS5228. 16S amplicon sequences are deposited in the National Center for Biotechnology Information (NCBI) Sequence Read Archive (SRA) under project number PRJNA769790. Publicly available metagenomes were accessed from NCBI SRA project number PRJNA385855 101 and newly deposited historical metagenomes from NCBI SRA project number PRJNA769790. CTD data are deposited in the Biological and Chemical Oceanography Data Management Office (BCO-DMO) at http://lod.bco-dmo.org/id/dataset/861266 for BIOS-SCOPE cruises, and at http://lod.bco-d-m.org/id/dataset/3782 for BATS cruises. Code for processing the raw mass spectrometry data is available at https://github.com/KujawinskiLaboratory/UntargCode. Code for processing the raw amplicon data is available at https://github.com/lbolanos32/NAAMES_2020. Code for PhyloAssigner, analyzing the time-series, querying the metagenomes, and calculating metabolic redundancy is available under git project https://github.com/BIOS-SCOPE/FunctionalRedundancy.

## SUPPLEMENTAL MATERIAL

Supplemental material msystems.01540-25-s0001.pdf Supplemental figures and tables.

ASM does not own the copyrights to Supplemental Material that may be linked to, or accessed through, an article. The authors have granted ASM a non-exclusive, world-wide license to publish the Supplemental Material files. Please contact the corresponding author directly for reuse.
