# Supplementary material for: Seasonal patterns of DOM molecules are linked to microbial functions in the oligotrophic ocean
Source: mSystems. 2025 Dec 30;11(2):e01540-25. doi: 10.1128/msystems.01540-25 (PMC12911362; doi:10.1128/msystems.01540-25)
Supplement: Supplemental material — Supplemental figures and tables. [file msystems.01540-25-s0001.pdf]

AMERICAN SOCIETY FOR MICROBIOLOGY
Journals.ASM.org

Supplementary Material

Seasonal patterns of DOM molecules are linked to microbial functions in the oligotrophic ocean

Erin L. McParland,[1,2*] Fabian Wittmers,[3,4] Luis M. Bolaños,[5] Craig A. Carlson,[6,7] Ruth Curry,[7] Stephen J. Giovannoni,[8] Michelle Michelsen,[5] Rachel J. Parsons,[7] Melissa C. Kido Soule,[2] Gretchen J. Swarr,[2] Ben Temperton,[5] Kevin Vergin,[9] Alexandra Z. Worden,[3,4] Krista Longnecker,[2] Elizabeth B. Kujawinski[2]

[1]College of Earth, Ocean, and Atmospheric Sciences, Oregon State University, Corvallis, OR, USA

[2]Department of Marine Chemistry and Geochemistry, Woods Hole Oceanographic Institution, Woods Hole, MA, USA

[3]Ocean EcoSystems Biology Unit, RD3, GEOMAR Helmholtz Centre for Ocean Research Kiel, Kiel, Germany

[4]Marine Biological Laboratory, Woods Hole, MA, USA

[5]Department of Biosciences, University of Exeter, Exeter, United Kingdom

[6]Department of Ecology, Evolution, and Marine Biology, Marine Science Institute, University of California, Santa Barbara, Santa Barbara, CA, USA

[7]Bermuda Institute of Ocean Sciences, Global Futures Laboratory, Arizona State University, St George's, Bermuda

[8]Department of Microbiology, Oregon State University, Corvallis, OR, USA

[9]Microbial DNA Analytics, Phoenix, OR, USA

[*]Address correspondence to erin.mcparland@oregonstate.edu

Supplementary Figures

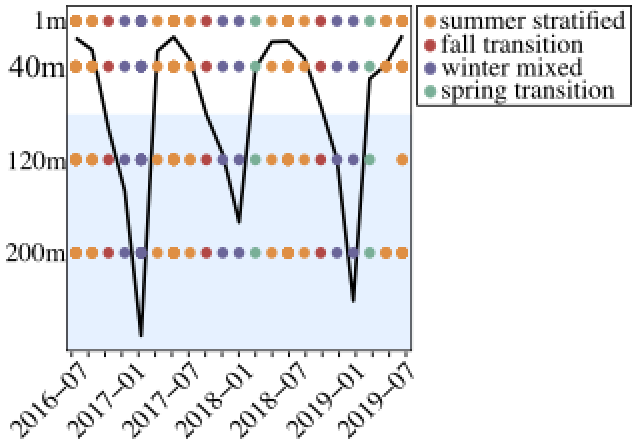

FIG S1 Spatiotemporal coverage of samples collected in the three-year time-series. Samples are colored by the four major seasonal states of the water column at BATS. The black line reflects mixed layer depth. The blue color delineates the upper euphotic and upper mesopelagic zones.

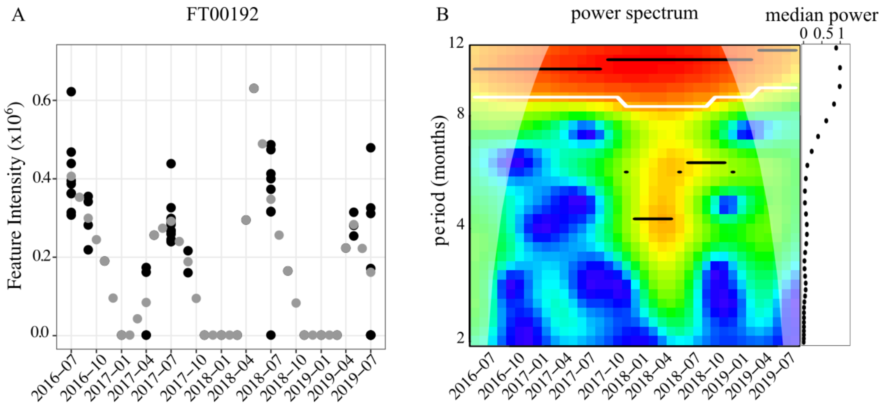

FIG S2 Example of the time-series wavelet analysis. (A) The original time-series (black) was almost uniformly sampled and contains multiple samples from the same month in some cases. The time-series was transformed to create a time-series with one sample per month (grey) (n = 37 samples). (B) Wavelet analysis was used to detrend the time-series as reflected by the resulting power spectrum. The side panel reflects the median power for each period. A higher median power indicates a better wavelet fit. In this example, the median power is highest for a period >11 months and was therefore considered to be a seasonal time-series.

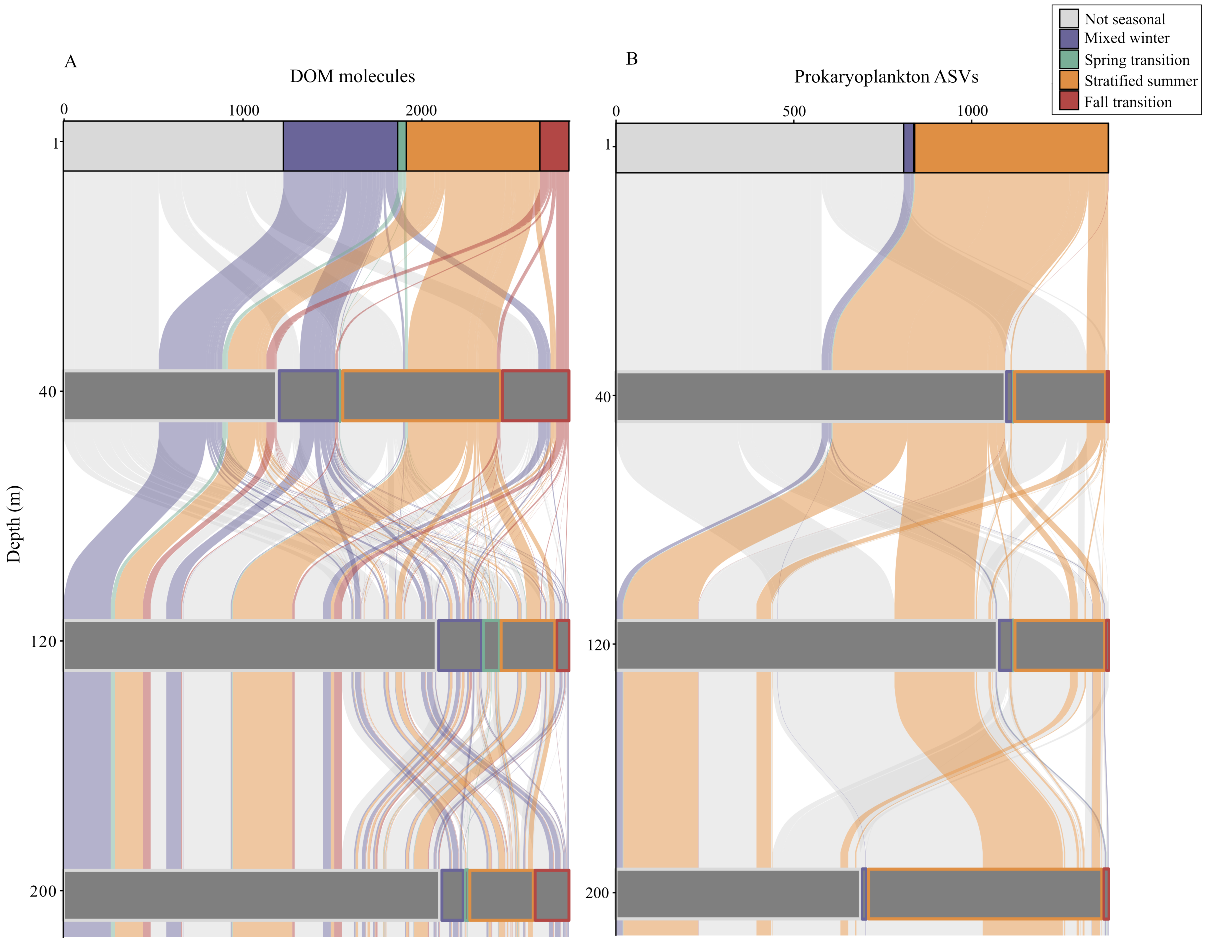

FIG S3 Alluvial plots depicting the season of the 12-month period maximum of seasonal (A) DOM molecules and (B) prokaryoplankton across sampling depths. Horizontal boxes represent the total number of seasonal DOM molecules or prokaryoplankton. Box colors reflect the number of features that peaked in a given season at the respective sampling depth. Grey represents a feature that is not seasonal at that depth but becomes seasonal at another depth. The ribbon colors track the connectivity of seasonal DOM molecules or prokaryoplankton at 1 m through the water column.

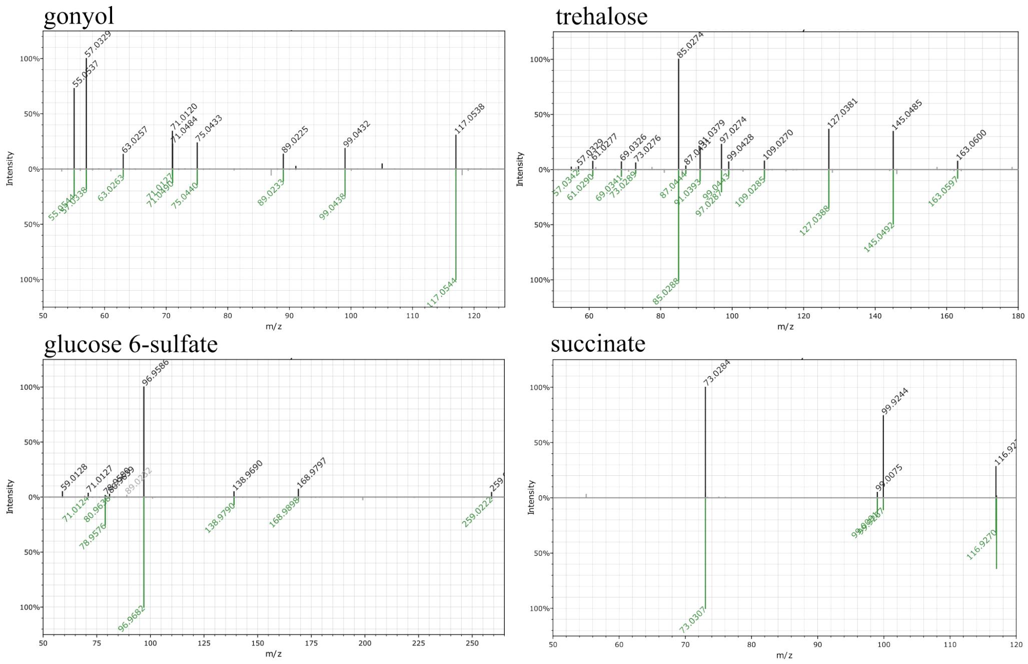

FIG S4 Mirror plot of MS2 spectra of putatively identified exometabolites from time-series (black) compared to the GNPS reference spectrum (green). In the glucose 6-sulfate mirror plot, the reference spectrum is of glucose 6-phosphate.

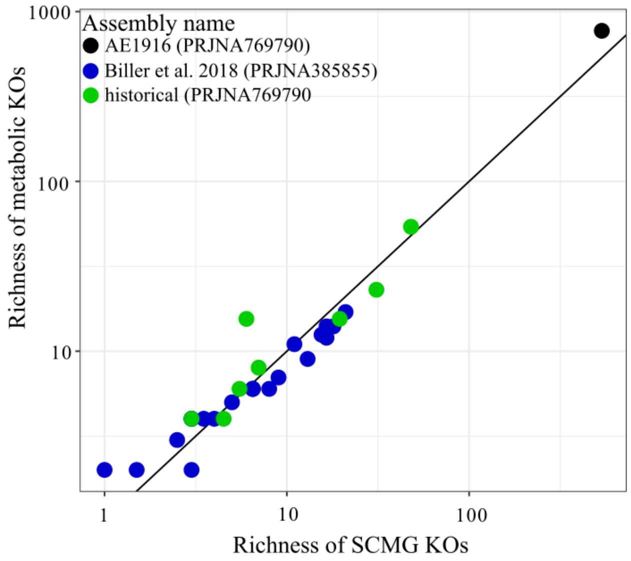

FIG S5 Median richness of all single copy marker gene (SCMG) KOs versus median richness of all trehalose and succinate KOs in surface samples (n = 28) of each assembly queried. Color reflects the dataset. The black line reflects a 1:1 relationship.

TABLE S1 Name of internal standard used in positive ionization mode, type of stable isotope label, detection in blanks (n = 15 total) or samples (n = 256), relative standard deviation in blanks or samples.

compound	label	detect blanks	detect samples	RSD blanks	RSD samples
leucine	D3	14	256	0.28	0.03
methionine	D3	15	256	0.07	0.06
phenylalanine	D8	15	256	0.03	0.07
proline	$^{13}C5,^{15}N1$	15	256	0.09	0.08
AMP	$^{15}N5$	15	253	0.20	0.12
biotin	D2	15	256	0.10	0.16
betaine	D11	11	254	0.66	0.14
pantothenate	$^{13}C3,^{15}N1$	0	248	-	0.20
lysine	D4	15	256	0.50	0.3
guanosine	D2	12	232	0.53	0.34
cysteine	D3	9	197	0.86	0.63

TABLE S2 Molecular formula, identification level as defined by the Metabolomics Standards Initiative, ionization mode, detected m/z, adduct, and retention time (minutes) of putatively identified exometabolites.

Compound	Formula	ID level	Ion mode	m/z	Adduct	Ret time
gonyol	$C_7H_{14}O_3S$	1	pos	179.0736	$[M+H]^+$	0.57
trehalose	$C_{12}H_{22}O_{11}$	1	pos	360.1504	$[M+NH4]^+$	0.61
glucose 6-sulfate (or galactose 6-sulfate)	$C_6H_{12}O_9S$	2	neg	259.0128	$[M-H]^-$	0.61
succinate	$C_4H_6O_4$	1	neg	117.0192	$[M-H]^-$	1.24

TABLE S3 Metagenome assemblies queried for functional redundancy analyses.

	Assembly name	Date	sample
1	hist PRJNA769790	1997-09-01	108_0
2	hist PRJNA769790	1998-02-01	113_0
3	hist PRJNA769790	1999-11-01	134_0
4	hist PRJNA769790	2000-01-01	136_0
5	hist PRJNA769790	2000-03-01	138_0
6	hist PRJNA769790	2001-08-01	155_0
7	hist PRJNA769790	2002-05-01	164_0
8	Biller PRJNA385855	2003-03-22	SRR5720238
9	Biller PRJNA385855	2003-04-22	SRR5720327
10	Biller PRJNA385855	2003-05-20	SRR5720283
11	Biller PRJNA385855	2003-07-15	SRR5720235
12	Biller PRJNA385855	2003-08-12	SRR5720286
13	Biller PRJNA385855	2003-10-07	SRR5720332
14	Biller PRJNA385855	2003-11-04	SRR5720276
15	Biller PRJNA385855	2003-12-02	SRR5720262
16	hist PRJNA769790	2003-03-01	174A_0
17	Biller PRJNA385855	2004-01-27	SRR5720338
18	Biller PRJNA385855	2004-03-23	SRR5720337
19	Biller PRJNA385855	2004-04-21	SRR5720256
20	Biller PRJNA385855	2004-05-18	SRR5720257
21	Biller PRJNA385855	2004-06-15	SRR5720260
22	Biller PRJNA385855	2004-08-17	SRR5720321
23	Biller PRJNA385855	2004-09-14	SRR5720251
24	Biller PRJNA385855	2004-10-13	SRR5720307
25	Biller PRJNA385855	2004-11-12	SRR5720278
26	Biller PRJNA385855	2004-12-08	SRR5720342
27	Biller PRJNA385855	2009-07-14	SRR6507279
28	AE1916 PRJNA769790	2019-07-09	5_1_S27

TABLE S4 List of succinate and trehalose KOs queried in all surface metagenomes for trehalose and succinate production or consumption.

compound	group	KO	enzyme
succinate	dehydrogenase	K00135	succinate-semialdehyde/glutarate-semialdehyde dehydrogenase
succinate	dehydrogenase	K00139	succinate-semialdehyde dehydrogenase
succinate	dehydrogenase	K17761	succinate-semialdehyde dehydrogenase, mitochondrial
succinate	dehydrogenase	K08324	succinate-semialdehyde dehydrogenase
succinate	succinyl CoA synthetase	K01902	succinyl-CoA synthetase alpha subunit
succinate	succinyl CoA synthetase	K01899	succinyl-CoA synthetase alpha subunit
succinate	glutarate oxidoreductase	K15737	glutarate dioxygenase
succinate	succinyl CoA transferase	K18118	succinyl-CoA:acetate CoA-transferase
succinate	dehydrogenase	K00244	succinate dehydrogenase flavoprotein subunit
succinate	dehydrogenase	K00234	succinate dehydrogenase (ubiquinone) flavoprotein subunit
succinate	dehydrogenase	K00239	succinate dehydrogenase flavoprotein subunit
trehalose	glycosyltransferase	K13057	trehalose synthase
trehalose	glucosyltransferase	K05343	maltose alpha-D-glucosyltransferase / alpha-amylase
trehalose	trehalohydrolase	K01236	maltooligosyltrehalose trehalohydrolase
trehalose	phosphatase	K01087	trehalose 6-phosphate phosphatase
trehalose	trehalase	K22934	alpha,alpha-trehalase
trehalose	trehalase	K01194	alpha,alpha-trehalase
trehalose	phosphorylase	K05342	alpha,alpha-trehalose phosphorylase
